# Spillover of *Mycobacterium bovis* from Wildlife to Livestock, South  Africa

**DOI:** 10.3201/eid2103.131690

**Published:** 2015-03

**Authors:** Jolly Musoke, Tiny Hlokwe, Tanguy Marcotty, Ben J.A. du Plessis, Anita L. Michel

**Affiliations:** University of Pretoria, Onderstepoort, South Africa (J. Musoke, T. Hlokwe, T. Marcotty, A.L. Michel);; Agricultural Research Council–Onderstepoort Veterinary Institute, Onderstepoort (T. Hlokwe);; Institute of Tropical Medicine, Antwerp, Belgium (T. Marcotty);; Animal Health Services, Ehlanzeni South, South Africa (B.J.A. du Plessis)

**Keywords:** bovine tuberculosis, Mycobacterium bovis, wildlife tuberculosis, Kruger National Park, wildlife–livestock–human interface, South Africa, tuberculosis and other mycobacteria, zoonoses, spillback, spillover, bacteria, tuberculsos

## Abstract

During August 2012–February 2013, bovine tuberculosis was detected in communal livestock bordering the Greater Kruger National Park Complex (GKNPC) in South Africa. Using spacer oligonucleotide and variable number tandem repeat typing, we identified the *Mycobacterium bovis* strain endemic in GKNPC wildlife. Our findings indicate bovine tuberculosis spillover from GKNPC wildlife to neighboring livestock.

Bovine tuberculosis is an infectious disease caused by *Mycobacterium bovis*. The wide host range of the pathogen comprises humans and domestic and wild animals. Great strides in controlling bovine tuberculosis have drastically reduced its prevalence in livestock and humans, particularly in industrialized countries. However, in developing countries in southern Africa and elsewhere, bovine tuberculosis remains a challenge to animal health because of a total or partial lack of bovine tuberculosis control, limited by a lack of funds (*1*,*2*). The control and/or elimination of bovine tuberculosis in both developing and industrialized countries can be complicated by wildlife reservoirs of the disease, which pose a threat of re-infection in livestock (*3*). In sub-Saharan Africa, particularly South Africa and Uganda, African buffalos (*Syncerus caffer*) serve as wildlife reservoirs of bovine tuberculosis; in Zambia, lechwe antelopes (*Kobus leche Kafuensis*) have been identified as wildlife reservoirs (*4*,*5*). New reports have suggested greater kudu (*Tragelaphus strepsiceros*) and common warthog (*Phacochoerus africanus*) as potential wildlife reservoirs of bovine tuberculosis (*4*).

*M. bovis* is endemic in buffaloes and has spilled into other wildlife species, particularly in the Kruger National Park (KNP) and adjacent game reserves that form part of the Greater Kruger National Park Complex (GKNPC) in South Africa (*6*,*7*). Except for data from sporadic regulatory bovine tuberculosis surveillance activities in cattle adjacent to the GKNPC, no data exist on the transmission of bovine tuberculosis from the GKNPC, where it is endemic, into livestock in neighboring communities (*3*). Because of the potentially negative implications of livestock–wildlife interactions on livestock and human health, the presence and role of zoonotic diseases in these communities needs to be investigated (*5*). We report on an investigation into the status and genotype of bovine tuberculosis in livestock in rural communities bordering the bovine tuberculosis–endemic GKNPC.

## The Study

**Figure 1:**
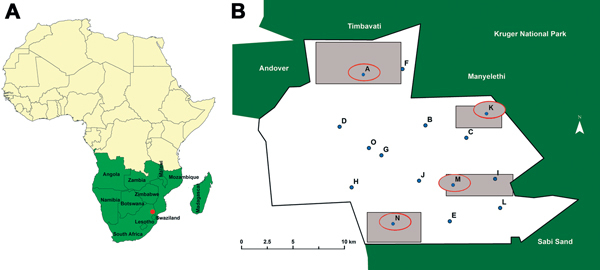
Location of study area (A, red dot) and location of dip tanks (B) in study of bovine tuberculosis transmission, Greater Kruger National Park Complex, South Africa, August 2012–February 2013. Parentheses used below indicate the shortest distance between individual dip tanks and the game fence, as follows: dip tank A (3.1 km), B (3 km), C (4.2 km), D (7.3 km), E (2.3 km), F (1 km), G (6.1 km), H (5.8 km), I (0.5 km), J (6 km), K (1.2 km), L (1 km), M (4.3 km), N (2 km), O (6.4 km). Blue dots indicate dip tanks sampled; red circles indicate dip tanks at which bovine tuberculosis–positive cattle were detected. Gray boxes indicate observed cattle grazing range for dip tanks at which bovine tuberculosis was detected.

The study was conducted in a rural community under the Mnisi Tribal Authority. The community is situated in Mpumalanga Province, South Africa, and borders the GKNPC in the west and 1 private game reserve (Figure 1). We constructed maps for this study using ArcGIS version 10.2 (http://www.arcgis.com). The KNP and private game reserves are fenced and have buffer zones established by double fencing (*8*).

Cattle farming, an essential part of the livelihood of the Mnisi community, is practiced primarily on a communal basis. Approximately 12,000 cattle live within the Mnisi area with 15 dip tanks, where the national government provides veterinary extension services and which represent epidemiologic units for sampling (Figure 1). Farmers in the study area are assigned and registered to a particular dip tank by the agricultural authorities by a stock card system. On a stock card, number of cattle, births/deaths, and animal movement are recorded. For this study, a dip tank is considered a whole herd because animals in a particular dip tank interacted extensive during grazing and dip tank inspections, and usually 1 herdsman was in charge of multiple stock cards.

During August 2012–February 2013, a total of 1,166 cattle at the 15 dip tanks in the study area were tested for bovine tuberculosis by using the comparative intradermal tuberculin test (CIDT). Animals selected for testing were chosen randomly from a list of stock cards at each dip tank. From each stock card chosen, we selected 10% of registered animals; however, a minimum of 2–3 animals per stock card were tested. All stock card owners willing to participate were included to reach a target of 10% of cattle assigned to each dip tank. We calculated the bovine tuberculosis status and 95% CI per dip tank assuming a binomial distribution of the data (Table 1).

**Table 1:** Status of bovine tuberculosis detected by using comparative intradermal tuberculin testing at 15 dip tanks, Greater Kruger National Park Complex, South Africa, August 2012–February 2013

Dip tank	No. cattle	No. cattle tested (%)	Test results	
			Inconclusive, no. animals (%; 95% CI)	Positive, no. animals (%; 95% CI)
A	1,648	178 (10.8)	6 (3.4; 1.5–7.3)	1 (0.6; 0.1–3.9)
B	556	55 (9.9)	3 (5.5; 1.8–15.6)	0 (0; 0–5.3)
C	963	104 (10.8)	2 (1.9; 0.5–7.4)	0 (0;0–2.8)
D	706	72 (10.2)	3 (4.2;1.4–12.1)	0 (0;0–4.1)
E	585	82 (14.0)	1 (1.2; 0.2–8.1)	0 (0; 0–3.6)
F	786	75 (9.5)	0 (0; 0–3.9)	0 (0; 0–3.9)
G	1,092	86 (7.9)	3 (3.5; 1.1–10.3)	0 (0;0–3.4)
H	709	70 (9.9)	3 (4.3; 1.4–12.5)	0 (0; 0.0–4.2)
I	850	75 (8.8)	1 (1.3; 0.2–8.9)	0 (0; 0–3.9)
J	545	48 (8.8)	1 (2.1; 0.3–13.4)	1 (2.1; 0.3–13.4)
K	436	49 (11.2)	3 (6.1; 2–17.3)	1 (2; 0.3–13.1)
L	812	79 (9.7)	2 (2.5; 0.6–9.6)	0 (0; 0.0–3.7)
M	903	50 (5.5)	1 (2; 0.3–12.9)	0 (0; 0.0–5.8)
N	1,298	83 (6.4)	2 (2.4; 0.6–9.1)	1 (1.2; 0.2–8.1)
O	943	60 (6.4)	1 (1.7; 0.2–10.9)	0 (0; 0–4.9)
Total	12,832	1,166 (9.1)	32	4

A whole-blood interferon-γ (IFN-γ) assay was performed as an ancillary test to the CIDT on all 4 CIDT-positive cattle and all 5 cattle with inconclusive reactions (defined as a difference between the bovine and avian increase in skin-fold thickness of >3 mm) (Table 2). Among the 9 cattle, 4 animals were classified as bovine tuberculosis reactors on the basis of the IFN-γ assay response (*9*), 1 each in 4 of the 15 dip tanks (Figure 1).

**Table 2:** CIDT results, whole-blood IFN-γ results, pathologic examination, and culture results of cattle tested for *Mycobacterium bovis* infection, Greater Kruger National Park Complex, South Africa, August 2012–February 2013*

Animal ID	Bovine bias†	CIDT	IFN-γ assay	Macropathology	Culture
N1	8.2	Positive	ND	NVL	*M. bovis*
A1	5.5	Positive	Positive	Multiple lesions in mediastinal and bronchial lymph nodes; single lung lesion	*M. bovis*
J1	5.4	Positive	Negative	NA	NA
K1	4.8	Positive	Positive	Multiple lesions in bronchial, lumbar and renal lymph nodes	*M. bovis*
K1 calf	ND	ND	ND	Single lung lesion	*M. bovis*
MI	3.8	Inconclusive	Positive	NVL	*M. bovis*
OI	3.8	Inconclusive	Negative	NA	NA
HI	3.5	Inconclusive	Negative	NA	NA
AI	3.5	Inconclusive	Negative	NA	NA
GI	3.1	Inconclusive	Negative	NA	NA

*CIDT, comparative intradermal tuberculin test results; ID, identification; IFN-γ, interferon-γ; NA, not applicable (animals were not slaughtered); ND, not done because of poor sample quality; NVL, nonvisible lesions. †Difference in skin thickness increase elicited by bovine and avian purified protein derivatives.

Animals classified as bovine tuberculosis reactors were purchased and slaughtered. These animals included a 1-month-old calf born to a CIDT- and IFN-γ assay–positive cow (animal no. K1). Standard sets of tissue samples were collected and cultured as previously described (*10*). Pathologic examination and culture results are shown in Table 2.

**Figure 2:**
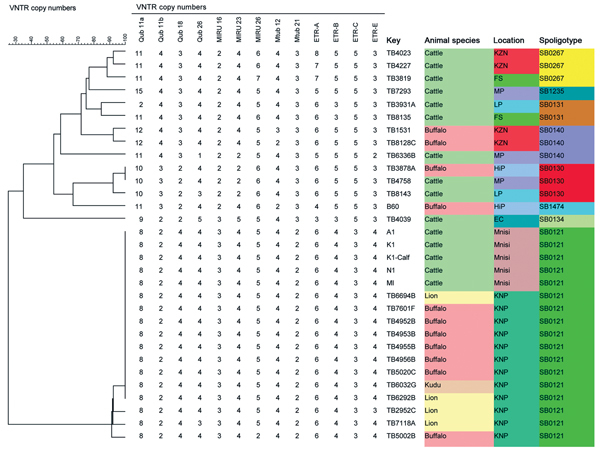
Dendogram depicting the genetic homology between isolates obtained in study of bovine tuberculosis transmission in the Greater Kruger National Park Complex during August 2012–February 2013 and from other outbreaks in South Africa. Colors differentiate the isolates. EC, Eastern Cape; FS, Free State; HiP, Hluhluwe iMofolozi Game Reserve; KNP, Kruger National Park (current study area [Mnisi]); KZN, Kwa-Zulu Natal; LP, Limpopo; MP, Mpumalanga; VNTR, variable number tandem repeat typing.

*M. bovis* was isolated from the 5 animals, and the isolates were characterized by using spacer oligonucleotide typing (spoligotyping) (*11*). Spoligotypes were named according to the *M. bovis* spoligotype database (http://www.mbovis.org). Variable number tandem repeat (VNTR) typing of the isolates was performed as previously described (*12*). Spoligotyping showed a single *M. bovis* spoligotype, SB 0121, in all isolates. NTR analysis using a 13-loci panel identified all isolates as the KNP VNTR 1, which constitutes the *M. bovis* outbreak strain responsible for the bovine tuberculosis epidemic in the KNP and the larger GKNPC (*12*) (Figure 2).

## Conclusions

We detected bovine tuberculosis in livestock directly bordering the GKNPC ecosystem. All 5 animals examined were infected with the same spoligotype and VNTR genotype of *M. bovis* as wildlife species in the adjacent GKNPC (*12*). This finding strongly suggests the spillover of *M. bovis* infection from wildlife to neighboring cattle (Figure 2). Alternatively, the KNP outbreak strain could have persisted in the area from which it entered the wildlife population of the GKNPC (*6*) and subsequently could have spread outside the KNP and reached the study area, a distance of ≈180 km. However, during 1996–2012, provincial State Veterinary Services of Mpumalanga tested a total of 96,806 head of cattle in this region of interest, which comprises the veterinary districts of Bushbuckridge (where the study area is located), Nsikazi (bordering the GKNPC in the west), and Nkomazi (south of KNP) using the CIDT as part of its regular bovine tuberculosis surveillance (B.J.A. du Plessis, unpub. data). No bovine tuberculosis reactors were detected in Bushbuckridge or in Nsikazi districts. In 3 unrelated outbreaks during 2009, 2010, and 2011 in the Nkomazi district, 1–3 bovine reactor animals were detected (B.J.A. du Plessis, unpub. data.). All outbreak strains were genotyped, and their spoligotypes and VNTR profiles differed from each other and from the *M. bovis* strain endemic to the GKNPC (results not shown). This information supports the hypothesis that bovine tuberculosis–infected cattle in our current study contracted *M. bovis* from neighboring wildlife in the GKNPC.

In conclusion, our study provides evidence that infected wildlife in the GKNPC constitute a risk factor for bovine tuberculosis infection of neighboring cattle, despite the separation of livestock and wildlife by a well-maintained disease control fence. These findings are of great concern, not only to livestock health and production in communities bordering the GKNPC but also to public health and to human livelihoods because of the zoonotic potential of bovine tuberculosis.
